# Mathematical models of targeted cancer therapy

**DOI:** 10.1038/sj.bjc.6603310

**Published:** 2006-10-10

**Authors:** L H Abbott, F Michor

**Affiliations:** 1 Program for Evolutionary Dynamics, Harvard University, Cambridge, MA 02138, USA; 2 Society of Fellows, Harvard University, Cambridge, MA 02138, USA

**Keywords:** targeted cancer therapy, mathematical biology, chronic myeloid leukaemia

## Abstract

Improved understanding of the molecular underpinnings of cancer initiation and progression has led to the development of targeted cancer therapies. The importance of these new methods of cancer treatment necessitates further research into the dynamic interactions between cancer cells and therapeutic agents, as well as a means of analysing their relationship quantitatively. The present review outlines the application of mathematical modelling to the dynamics of targeted cancer therapy, focusing particular attention on chronic myeloid leukaemia and its treatment with imatinib (Glivec).

The decision as to whether cells proliferate or die is dictated by complex networks of regulatory factors, stress signals and interactions with the surrounding microenvironment. Disruption of the normal regulation of cell division and differentiation plays a vital role in tumorigenesis (Vogelstein and Kinzler, 2001). Recent advances in the research examining these molecular causes of cancer offer the possibility of specifically targeting abnormal proteins and hence designing more efficient and effective therapy. ‘Targeted therapy’ refers to a new generation of cancer drugs that interact with specific molecular targets thought to lie at the heart of tumour growth or progression. The research into adequate targets is based on a detailed understanding of the genetic alterations driving tumorigenesis (Hanahan and Weinberg, 2000); this approach contrasts with the traditional strategy to discover nonspecific cytotoxic therapeutics. Table 1 provides a list of some currently used or researched targeted agents (Sawyers, 2004).

Targeting mutant kinases has proven to be highly successful in the treatment of cancers whose growth is acutely dependent on those proteins. Imatinib (Glivec) serves as an example of successful targeted therapy and is the topic of this review; it is used for the treatment of chronic myeloid leukaemia (CML), which is characterised by the BCR-ABL oncogene, and of gastrointestinal stromal tumours harbouring mutations in the c-Kit kinase. Two follow-up drugs to imatinib, AMN107 (nilotinib) and BMS-354825 (dasatinib), have been designed for even more efficient inhibition of BCR-ABL (Shah *et al*, 2004). Treatment of lung cancers with the epidermal growth factor receptor (EGFR) inhibitor, gefitinib (Iressa), is promising in patients carrying point mutations in the EGFR kinase domain (Couzin, 2002; Herbst *et al*, 2004). Avastin (bevacizamab) is a monoclonal antibody directed against the vascular endothelial growth factor and has been approved as a treatment of colon cancer (Marx, 2005). Avastin acts by inhibiting angiogenesis and might thus prove successful in treating many different tumour types.

A quantitative understanding of cancer biology requires the development of a mathematical framework capable of describing the fundamental principles governing tumour initiation and progression (Moolgavkar and Knudson, 1981; Knudson, 2001). The dynamics of tumorigenesis are determined by the same underlying principles that govern evolution: mutation and selection. Therefore, mathematical models can be used to study cancer initiation, progression and responses to therapy (Michor *et al*, 2004). Only when the dynamics of cancer cells during therapy are understood quantitatively can specific predictions be made about treatment success, cancer cell kinetics and failure of therapy owing to resistance. Mathematical models are therefore indispensable to a complete understanding of targeted therapy.

There have been only a few attempts made to examine the dynamic relationship between cancer cells and targeted therapeutic agents via mathematical modelling. Green *et al* (2001) developed a mathematical model for antibody-targeted therapy of colorectal cancer. They collected data on the *in vivo* distribution of antibodies against carcinoembryonic antigen and identified the most useful parameters for determining antibody localisation: the affinity for the antibody, the flow of the antibody through the tumour and the rate of elimination of the antibody from the tumour. Their model can help to optimally design antibody-based cancer therapy.

Wein *et al* (2002) proposed a mathematical model of the spatio-temporal dynamics of a brain tumour treated with a specific cytotoxic agent. They derived a formula for the tumour-cure probability given specific parameters regarding tumour characteristics, drug design and drug delivery. They then determined the required circumstances within which targeted therapy can be effective.

Iwasa *et al* (2003) and Michor *et al* (2006) examined the probability of resistance to targeted cancer therapy with a model based on multi-type branching processes. They calculated the escape dynamics for arbitrary mutation networks necessary to confer resistance, and extended this to cover any possible fitness landscape. They determined the probability of the success and failure of biomedical intervention against rapidly evolving cells.

Charusanti *et al* (2004) presented a mathematical model of signalling events in CML cells. They examined the effects of Glivec on the autophosphorylation of the BCR-ABL oncoprotein and subsequent signalling through the Crkl pathway, and predicted a minimal concentration for drug effectiveness. The model suggests that cellular drug clearance mechanisms reduce the efficacy of Glivec in blast crisis cells, and that these resistance mechanisms might be present from the onset of disease.

Araujo *et al* (2005) used mathematical modelling to investigate combination therapy in which multiple nodes in a signal transduction pathway are targeted simultaneously with specific inhibitors. They demonstrated that the attenuation of signalling is significantly enhanced when several upstream processes are inhibited, and that this weakening is most pronounced in signals downstream of serially connected targets.

Komarova and Wodarz (2005a, 2005b) presented a mathematical framework to study the emergence of resistance in cancers treated with targeted small-molecule drugs. They considered a stochastic dynamical system based on the turnover rate of tumour cells and the rate at which resistant mutants arise, and they found that resistance develops mainly before the start of treatment and, for cancers with high turnover rates, that combination therapy is less likely to yield an advantage over single-drug therapy.

There exists a large volume of literature concerning mathematical models of antiviral therapy, and many of the ideas arising in this context can be applied to targeted cancer therapy (Nowak and May, 2000).

Michor *et al* (2005) designed a mathematical model to analyse the *in vivo* kinetics of CML during treatment with the targeted agent imatinib (Glivec). The following discussion will outline the approach and importance of this promising method of cancer treatment while emphasising the need for further investigations into the mathematical models capable of describing clinical responses to these therapies.

## DYNAMICS OF CML

Chronic myeloid leukaemia is a blood cancer characterised by excessive numbers of granulocytes, erythrocytes and platelets in peripheral blood (Sawyers, 1999). The molecular hallmark of CML is the Philadelphia (Ph) chromosome: a reciprocal 9;22 translocation generating a fusion oncogene, BCR-ABL. The Ph chromosome arises in a haematopoietic stem cell and renders the cell's growth and survival independent of cytokines (Gishizky and Witte, 1992). This proliferative independence is then passed along to each daughter cell, which eventually leads to the clinical manifestations of CML. The disease generally progresses through three phases: a benign chronic phase that may last several years untreated, followed by an accelerated phase which terminates in the third, rapidly fatal phase known as the blast crisis (Sawyers, 1999; Deininger *et al*, 2005).

Before the introduction of imatinib mesylate (Glivec) as a targeted therapy for CML, treatment options were relatively unsuccessful and often costly or of limited applicability. Allogeneic bone marrow transplant resulted in event-free survival rates between 40 and 70%; however, only 20–25% of CML patients were eligible for related allogeneic transplant owing to the need for a matching bone marrow donor (Kantarjian *et al*, 1996). Thus, most patients were initially treated with hydroxyurea, busulfan or interferon alpha, the latter of which attempted to use the body's natural defence proteins to combat the disease. Intensive chemotherapy has also been attempted as a treatment regimen, generally leading to limited cytogenetic remission in 60–70% of patients. Lastly, autologous stem cell transplantation represents another treatment option offering a 4- to 5-year survival rate of 56–70% following the transplant (Kantarjian *et al*, 1996).

The revolution in CML treatment came about with the discovery of imatinib mesylate (Glivec) as a targeted chemotherapeutic agent (Druker *et al*, 2001). This compound was demonstrated to be effective in all stages of the disease (Kantarjian *et al*, 2002; Sawyers *et al*, 2002). Imatinib binds competitively with ATP to BCR-ABL and blocks its abnormal signalling. It selectively inhibits the proliferation of BCR-ABL-positive cell lines. However, acquired resistance to imatinib develops in a substantial fraction of patients. In 70–80% of these cases, acquired resistance is caused by point mutations in the ABL kinase domain (Gorre *et al*, 2001). So far, about 40 different point mutations have been discovered, each of which is sufficient to cause resistance to imatinib (Branford *et al*, 2003a). Of those patients who start imatinib in the early chronic, late chronic and accelerated phase, respectively, 12, 32 and 62% develop detectable resistance within 2 years of treatment (Branford *et al*, 2003b). Here, early chronic phase refers to patients who commenced imatinib within 1 year of diagnosis.

Imatinib stands as a prime example of what may be possible due to the burgeoning rise of targeted cancer therapies. However, imatinib fails to eliminate residual disease which has been shown to be part of the bone marrow compartment (Bhatia *et al*, 2003; Muller *et al*, 2003; Chu *et al*, 2005). Several important questions remain: What are the dynamics of relapse due to imatinib resistance? Can imatinib eradicate leukaemic stem cells? What effect does imatinib exert on different subpopulations of leukaemic cells? How can combination therapy be optimally administered? The answers to such questions are crucial to the appropriate treatment of patients diagnosed with CML, and they require a quantitative means of analysing the available data. Mathematical modelling offers just such an analytical tool.

### The data

**Figure 1:**
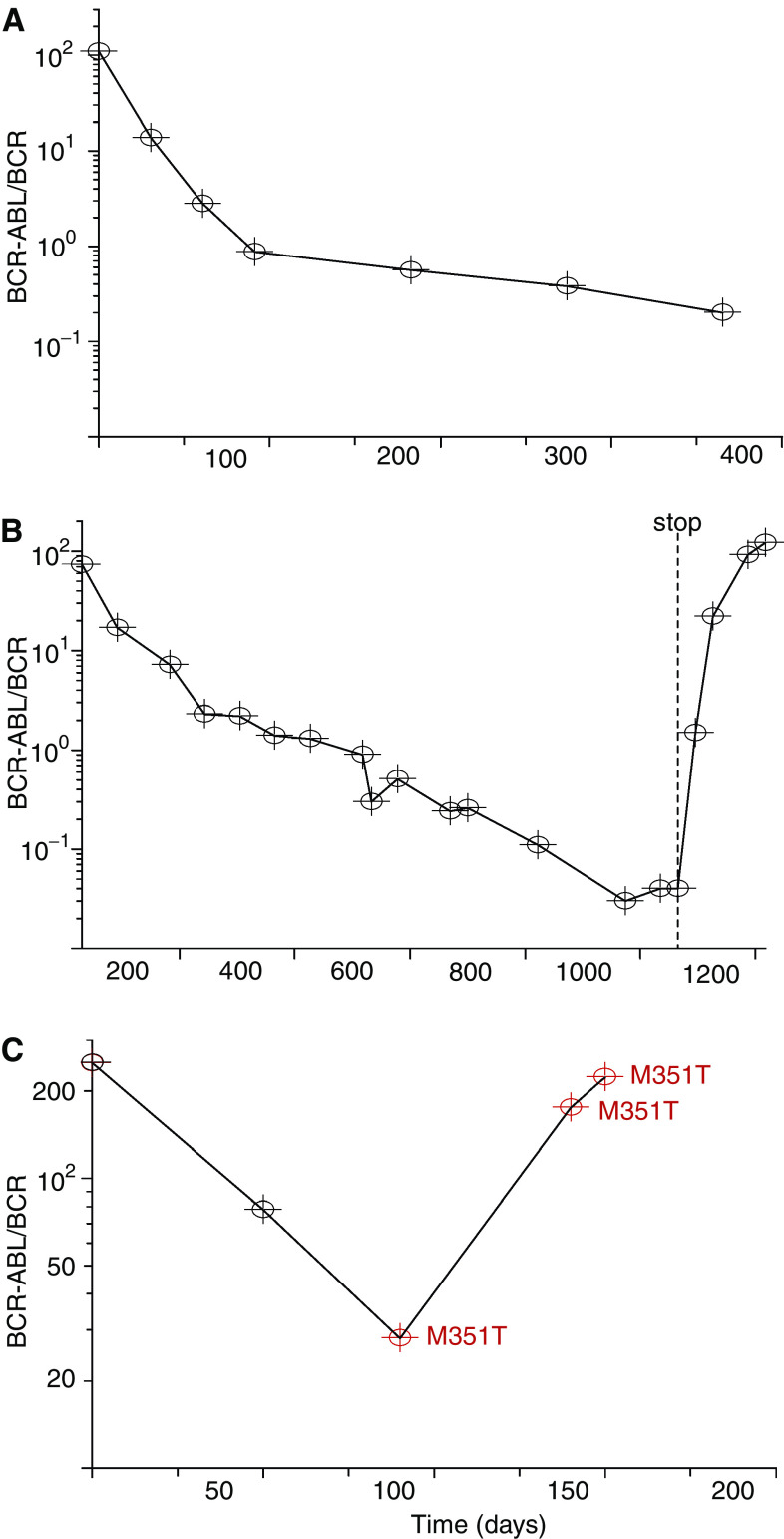
Chronic myeloid leukaemia patient data. The figure shows the leukaemic cell burden in a patient without resistance (**A**), in a patient whose therapy is stopped (**B**) and in a patient developing resistance (**C**). Imatinib therapy commences at day 0, and the percentage of leukaemic cells in peripheral blood is measured by quantitative PCR of the BCR-ABL oncogene normalised by the values of BCR. (**A**) Upon initiation of imatinib treatment, the leukaemic cell burden declines bi-phasically. The first slope reflects the depletion of differentiated cancer cells and the second slope reflects the depletion of leukaemic progenitors. (**B**) If therapy is stopped, the leukaemic cell load returns to levels at or beyond pretreatment baseline because imatinib is not capable of decreasing the abundance of leukaemic stem cells. (**C**) Resistance evolves in many patients after a variable period of successful therapy. The patient shown developed the resistance mutation M351T (methionine-threonine substitution at position 351) before day 100 of therapy. Figure adapted from Michor *et al* (2005).

In Michor *et al* (2005), we analysed data from 169 CML patients followed over 12 months of treatment with imatinib. The disease burden was monitored by quantitative PCR of the BCR-ABL oncogene, normalised by the value of BCR to compensate for the efficiency of reverse transcription and variations in RNA quality. Most of the patients show a biphasic decline of the leukaemic cell burden (Figure 1A). The average of the first slope is 0.05±0.02 and suggests that there is a subpopulation of cancer cells that has a mean lifespan of 1/0.05=20 days during imatinib therapy. The average of the second slope is 0.008±0.004, suggesting that another subpopulation of cancer cells has a mean lifespan of 1/0.008=125 days during treatment. Imatinib leads to a 5000-fold decline in the leukaemic cell count over the first 12 months of therapy.

With these data, we can quantify the CML kinetics *in vivo*. We know from the biology of the haematopoietic system (Hoffbrand *et al*, 1999; Beutler *et al*, 2000; Shizuru *et al*, 2005) that terminally differentiated cells have a lifespan of approximately 1 day. The first slope seen in the data suggests that there is a subpopulation, the differentiated leukaemic cells, that have an average lifespan of 20 days, and the second slope suggests that another subpopulation, the leukaemic progenitors, have a lifespan of 125 days during imatinib therapy.

We used data from patients who discontinued treatment to infer the effect of imatinib on leukaemic stem cells. Despite continuous therapy of up to 3 years, the leukaemic cell load rapidly returns to levels at or beyond pretreatment baseline upon discontinuation of imatinib (Figure 1B). This rapid resurgence of leukaemic cells after cessation of therapy is due to the fact that imatinib inhibits the production of terminally differentiated cancer cells 5000-fold; hence, discontinuation of therapy leads to a 5000-fold increase in the production of leukaemic cells. The level the leukaemic cell load reaches after discontinuation signifies the behaviour of the cell population that is driving the disease: the leukaemic stem cells. If the cell number settles at levels well below pretreatment baseline, then leukaemic stem cells were depleted by therapy. If the levels settle at or beyond the baseline as seen in our patients, then leukaemic stem cells are not depleted by imatinib and this chemotherapeutic cannot cure the disease.

Patients can relapse despite continuous therapy owing to the evolution of resistance (Figure 1C). Thirty patients in our database developed resistance that could be detected after a variable duration of successful therapy.

### The model

We developed a mathematical model that describes four subpopulations of the haematopoietic system, as suggested by the data: stem cells, progenitors, differentiated cells and terminally differentiated cells. This differentiation hierarchy applies to normal, leukaemic and resistant cells. The abundances of normal stem cells, progenitors, differentiated and terminally differentiated cells are denoted by *x*_0_, *x*_1_, *x*_2_ and *x*_3_; the respective abundances of leukaemic cells are denoted by *y*_0_, *y*_1_, *y*_2_ and *y*_3_, and those of resistant cells by *z*_0_, *z*_1_, *z*_2_ and *z*_3_. Only stem cells have unlimited self-renewal propensities and can continually replenish the complete differentiation hierarchy. The BCR-ABL oncogene is present in all leukaemic cells and leads to a slow clonal expansion of leukaemic stem cells. Furthermore, it increases the rate at which leukaemic stem cells produce progenitors and differentiated cells (Michor *et al*, 2005). The rate constants for the production of progenitors, differentiated cells and terminally differentiated cells are given by *a*, *b* and *c*, with appropriate indices to distinguish between healthy, leukaemic and resistant cell lineages. Leukaemic stem cells mutate at rate *u* per cell division to give rise to resistant stem cells. Stem cells die at rate *d*_0_, progenitors at rate *d*_1_, differentiated cells at rate *d*_2_ and terminally differentiated cells at rate *d*_3_ per day. Hence, the basic model is given by



Density dependence is achieved by an appropriate declining function, λ(*x*_0_). Normal cells remain at their equilibrium abundances, *x*_0_^*^=*x*_0_, *x*_1_^*^=*a*_*x*_*x*_0_^*^/*d*_1_, *x*_2_^*^=*b*_*x*_*x*_1_^*^/*d*_2_ and *x*_3_^*^=*c*_*x*_*x*_2_^*^/*d*_3_. Initially, leukaemic stem cells grow exponentially following *y*_0_(*t*)=exp[(*r*_y_−*d*_0_)*t*]. Here, we ignore the evolution of resistance. BCR-ABL is assumed to increase the rate at which progenitors and differentiated cells are being produced; hence, *a*_*y*_>*a*_*z*_ and *b*_*y*_>*b*_*z*_. However, imatinib counteracts this effect by reducing those rates to *a*_*y*_′<*a*_*y*_ and *b*_*y*_′<*b*_*y*_ (Michor *et al*, 2005).

#### Model dynamics

**Figure 2:**
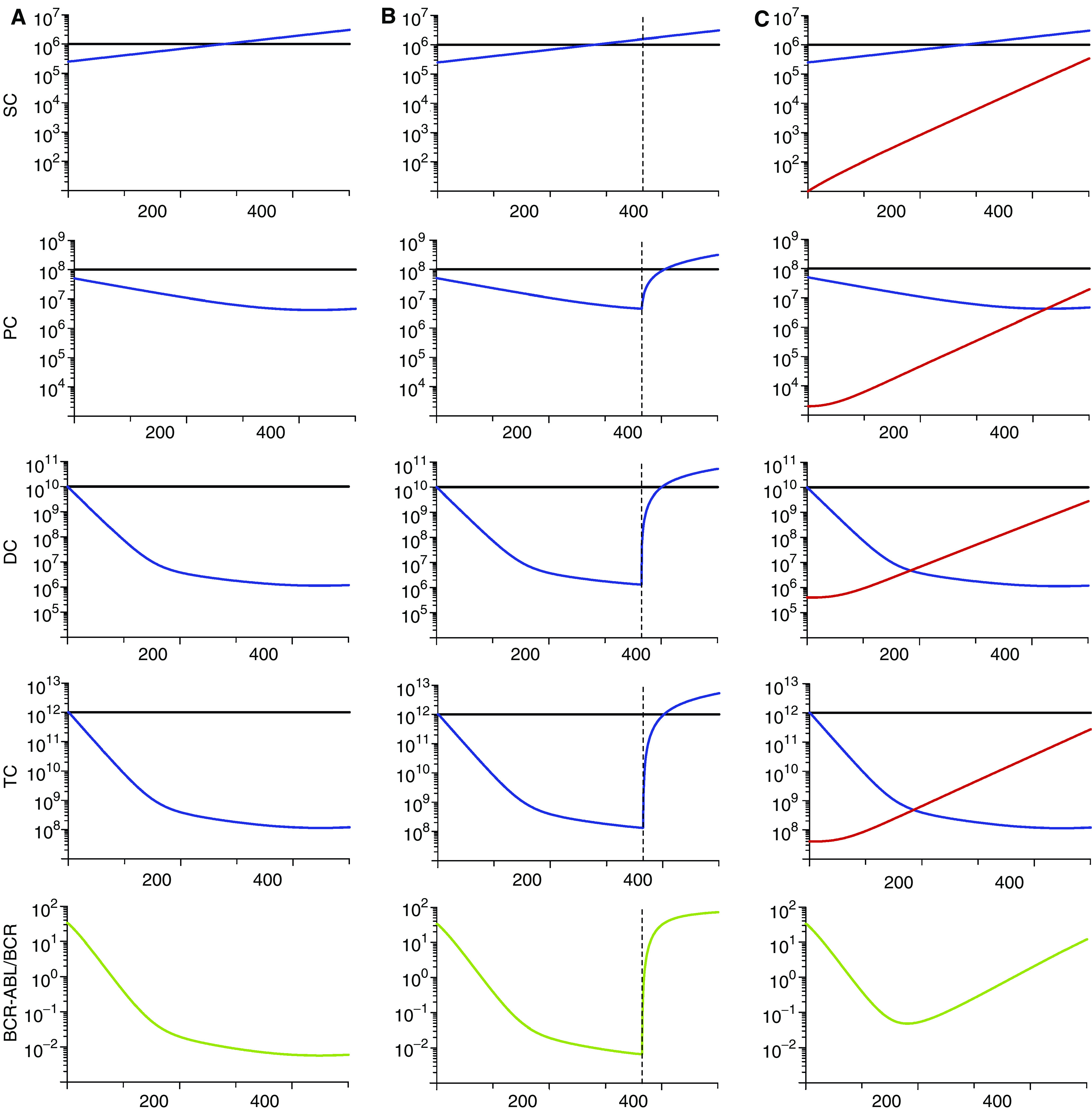
Model dynamics. The figure shows the dynamics of treatment without resistance mutations (**A**), when therapy is stopped (**B**), and with resistance mutations (**C**). The rows show the simulation output for stem cell dynamics (SC), progenitors (PC), differentiated (DC) and terminally differentiated cells (TC), and the ratio of BCR-ABL over BCR in percent (green curve). Wild-type cells are shown in black, leukaemic cells in blue and resistant leukaemic cells in red. (**A**) Imatinib therapy is started at day 0 and leads to a bi-phasic decline of the percentage of leukaemic cells. Leukaemic stem cells continue to expand at a slow rate. (**B**) Discontinuation of treatment (broken line) leads to a rapid relapse of leukaemic cells to levels beyond pretreatment baseline because leukaemic stem cells were not depleted during imatinib therapy. (**C**) Evolution of resistance leads to an increase in the percentage of cancer cells. Parameter values are *d*_0_=0.003, *d*_1_=0.008, *d*_2_=0.05, *d*_3_=1, *a*_x_=0.8, *b*_x_=5, *c*_x_=100, *r*_*y*_=0.008, *a*_*y*_=2*a*_*x*_, *b*_*y*_=2*b*_*x*_, *c*_*y*_=*c*_*x*_, *r*_*z*_=0.023. During therapy, we have *a*_*y*_′=*a*_*y*_/100, *b*_*y*_′=*b*_*y*_/750, *c*_*y*_′=*c*_*y*_, *a*_*z*_=*a*_*y*_′=*a*_*y*_, *b*_*z*_=*b*_*y*_′=*b*_*y*_ and *c*_*z*_=*c*_*y*_′=*c*_*y*_. In (c), we have *z*_0_(0)=10 and *u*=4.10^−8^. Figure adapted from Michor *et al* (2005).

Figure 2 summarises the dynamic features of the model. Figure 2A shows the simulation results of imatinib treatment without resistance starting at day 0. The leukaemic cell burden declines bi-phasically: terminally differentiated leukaemic cells decrease at their death rate, *d*_3_=1 per day, until they reach a steady state with differentiated leukaemic cells, then they track the latters' disease kinetics. Differentiated leukaemic cells decline at their death rate, *d*_2_=0.05 per day, until they reach an equilibrium with leukaemic progenitors. Progenitors decline at their death rate, *d*_1_=0.008 per day, until they reach an equilibrium with leukaemic stem cells. Stem cells themselves are not depleted by imatinib and continue to expand exponentially during therapy.

#### Stop therapy

If treatment is discontinued after 1 year (Figure 2B), then the leukaemic cell load rapidly rises to levels beyond pretreatment baseline because the leukaemic stem cell abundance did not decline during therapy. Our mathematical model (Michor *et al*, 2005) fitted to *in vivo* data suggests that imatinib may be incapable of depleting leukaemic stem cells; however, only three patients who discontinued imatinib therapy could be analysed because treatment is stopped very rarely. Once more data of stop patients becomes available, the theory might have to be revised, but the conclusion that leukemic stem cells cannot be depleted by imatinib can safely be drawn based on the available information. Additionally, previous work has shown that CML stem cells are insensitive to imatinib *in vitro* (Graham *et al*, 2002).

#### Resistance

Evolution of resistance leads to a relapse despite continuous therapy (Figure 2C). The probability of resistance can be calculated by considering an exponentially growing leukaemic stem cell population that produces resistant leukaemic cells at rate *u* per cell division. Upon reaching a certain abundance, *y*_0_, of leukaemic stem cells, the probability of having at least one resistant stem cell is given by *P*=1−exp (−*uy*_0_*σ*), where *σ*=(1+*s*)log(1+1/*s*) and *s*=(*a*_*y*_−*d*_0_)/*d*_0_. This calculation is based on the assumption that resistant stem cells have the same fitness as sensitive leukaemic stem cells. The formula for the probability of resistance for any fitness value and the expected number of resistant cells is derived in Iwasa *et al*, 2006.

The probability of resistance increases with the mutation rate, the number of leukaemic stem cells and the fitness of resistant stem cells. It also increases with the number of cell divisions that generated the leukaemia: the larger the number of cell divisions the higher the risk of mutations, and therefore the higher the probability of resistance. We can use the deterministic model to estimate the time until detection of resistance and treatment failure. These and other related calculations may be extremely helpful in determining when imatinib therapy ought to be combined with other therapies, providing a more accurate prognosis for the disease and directing future research and development efforts.

## CONCLUSION

Mathematical modelling of cancer progression and the evolution of resistance will prove invaluable in the ongoing struggle to develop new and more effective therapeutic strategies for the treatment of cancer. The model discussed above provides an improved understanding of treatment failure and success, as well as increased knowledge of the way therapy affects the cancer cell burden over time. The model also offers predictive power to the course the disease is likely to take, and therefore supplies both the physician and the researcher with some of the tools necessary to combat cancer cell proliferation to the greatest effect.

However, the research outlined above marks only the beginning of much needed explorations into the mathematical modelling of such systems. Targeted therapies will certainly play an increasingly vital role in the future of cancer treatment, particularly as we continue to accumulate knowledge of the molecular interactions responsible for tumorigenesis. Many of the dynamic interactions between targeted agents and cancer cells remain poorly understood. Although much research remains to be carried out in the realm of molecular biology, these relations will be greatly clarified by using a mathematical approach to *in vivo* therapeutic responses. As the era of targeted cancer therapy progresses and new drugs are developed, it becomes imperative that more energy be directed towards a quantitative approach to the analysis of therapeutic success and failure.

## Figures and Tables

**Table 1:** Targeted therapies and their agents

**Drug**	**Target**	**Function of target**	**Disease**
Imatinib (Gleevec) Nilotinib	Abl Kit PDFGR	Growth factor receptors	CML GIST HES CMML DFSP
Dasatinib	SFK/Abl Kit Lyn Sfc	Growth factor receptor Cell adhesion Migration and invasion	CML GIST Prostate cancer
Sunitinib malate (Sutent)	Multiple tyrosine kinases	Growth and angiogenesis	GIFT RCC
Gefitinib (Iressa) Erlotinib (Tarceva)	EGFR	Growth factor receptor	Lung cancer
Lapatinib (Tykerb)	EGFR ErbB2	Growth factor receptor Growth and differentiation	Solid tumours Lung cancer Breast cancer
Trastuzumab (Herceptin)	ErbB2	Growth and differentiation	Breast cancer
Bevacizamab (Avastin)	VEGF ligand	Angiogenesis	Colon cancer
Temsirolimus Everolimus	mTOR	Translation and cell division	Various cancers
Bortezombi (Velcade)	Proteasomes	Cell function and growth	Multiple myeloma
Oblimersen (Genasense)	BCL-2	Inhibition of apoptosis	Leukaemia Non-Hodgkin's lymphoma Solid tumours
PKC-412 NKB-518 CEP-701	FLT3	Growth factor receptor	AML
BAY 43-9006	VEGFR RAF	Angiogenesis Growth factor receptor	Kidney cancer Melanoma
SU-011248	VEGFR	Angiogenesis	Kidney cancer

AML=acute myeloid leukaemia; CML=chronic myeloid leukaemia; CMML=chronic myelomonocytic leukaemia; DFSP=dermatofibrosarcoma protuberans; EGFR=epidermal growth factor receptor; GIFT=gastrointestinal fibrous tumour; GIST=gastrointestinal stromal tumours; HES=hypereosinophilic syndrome; mTOR=mammalian target of rapamycin; PDGFR=platelet-derived growth factor receptor; RCC=renal cell carcinoma; SFK=Schistosoma mansoni Fer-like kinase; VEGF=vascular endothelial growth factor; VEGFR=vascular endothelial growth factor receptor.
